# ILC plasticity in intestinal immune barrier remodeling: current evidence and therapeutic implications for inflammatory bowel disease

**DOI:** 10.3389/fimmu.2026.1891742

**Published:** 2026-09-07

**Authors:** Ziqing Zhou, Ping Ling, Jinxin Wang, Xinrui Zhang, Yan Shen

**Affiliations:** 1 The Second Clinical Medical College, Zhejiang Chinese Medical University, Hangzhou, China; 2 The Second Affiliated Hospital of Zhejiang Chinese Medical University, Hangzhou, China

**Keywords:** ILC subpopulation plasticity, inflammatory bowel disease, innate lymphoid cells, intestinal immune barrier remodeling, intestinal mucosal immunity

## Abstract

Inflammatory bowel disease (IBD), which includes Crohn’s disease (CD) and ulcerative colitis (UC), is a group of chronic gastrointestinal inflammatory diseases characterized by persistent mucosal inflammation and intestinal immune barrier dysfunction. Innate lymphoid cells (ILCs) are key regulators of mucosal immunity; however, the contribution of their context-dependent plasticity to IBD-associated immune barrier remodeling remains incompletely defined. This narrative review summarizes the intestinal distribution and functional specialization of ILC subsets, their dysregulation in the IBD microenvironment, and the molecular signals that regulate ILC plasticity. Current evidence most strongly supports an ILC3-to-ILC1 transition in CD, whereas direct evidence for defined directional ILC plasticity in UC and for ILC2 lineage conversion in intestinal IBD remains limited. We also discuss the context-dependent roles of ILC2s in epithelial repair, inflammation, and fibrosis, as well as the functional reprogramming of ILC3s from barrier-protective to potentially pathogenic states. In addition, this review summarizes the cytokine, transcriptional, epigenetic, and metabolic mechanisms associated with ILC fate remodeling and discusses potential therapeutic strategies targeting these pathways. Overall, ILC plasticity may represent a disease- and tissue-specific mechanism contributing to intestinal immune barrier remodeling, although its causal role and therapeutic relevance require further validation in human IBD. These findings provide a framework for developing more precisely stratified and context-dependent therapeutic approaches for IBD.

## 1 Introduction

Inflammatory bowel disease (IBD) is a group of chronic, nonspecific inflammatory diseases affecting the gastrointestinal tract, primarily comprising Crohn’s disease (CD) and ulcerative colitis (UC) (1, 2).The age-standardized incidence of IBD worldwide rose from 4.22 cases per 100,000 people in 1990 to 4.45 cases per 100,000 in 2021, with the most significant increase observed in East Asia (annual growth rate of 2.89%). Among the 204 countries covered in the 2021 Global Burden of Disease study, China had the highest growth rate (2.93%) (3).

Intestinal immune barrier remodeling refers to the dynamic adjustment of epithelial architecture, immune-cell composition, the cytokine microenvironment, and tissue-repair pathways in response to physiological or pathological signals (4). Under physiological conditions, gut-associated lymphoid tissues, immune cells, and immune effector molecules cooperate to maintain immune homeostasis and protect against luminal antigens, thereby establishing the structural and functional basis for physiological barrier remodeling (2, 5). In IBD, this homeostasis is severely disrupted. Microbial dysbiosis, epithelial injury, and aberrant immune activation drive pathological remodeling of the intestinal immune barrier, characterized by altered immune-cell populations, dysregulated pro-inflammatory and anti-inflammatory signaling, and impaired epithelial repair and inflammation resolution (6–8). These changes ultimately lead to persistent mucosal inflammation, incomplete epithelial healing, and barrier dysfunction (2, 9, 10).

The patterns of pathological immune barrier remodeling differ between CD and UC. CD is commonly characterized by segmental and transmural inflammation, type 1 and type 17 immune activation, and progressive fibrostenotic remodeling (11). Human mucosal studies have additionally demonstrated an increased abundance of ILC1-like populations accompanied by a reduction in barrier-supportive NKp44^+^ ILC3 populations. In contrast, UC predominantly involves the colonic mucosa and is characterized by continuous superficial inflammation, goblet-cell dysfunction, mucus-barrier disruption, and cytokine-associated remodeling of epithelial and immune-cell states (12). Alterations in type 2 and type 3 ILC populations have also been reported. These disease-specific inflammatory environments may differentially shape the abundance, phenotype, and plasticity of intestinal ILC subsets.

Innate lymphoid cells (ILCs) are innate immune cells that reside predominantly in mucosal tissues, including the gastrointestinal tract, skin, and lungs, as well as in lymphoid organs associated with barrier immunity (13). The functions of immune effector cells, including ILCs, are not fixed; inflammatory and tissue-derived signals can induce reversible changes in subset phenotype and function, a process termed plasticity (14, 15). ILCs are key regulators of the intestinal immune barrier, but their subset-specific functions and dynamic plasticity during IBD-associated barrier remodeling remain incompletely understood. This narrative review first summarizes the intestinal biology of ILC subsets and then examines their dysregulation, plasticity, molecular regulation, and potential therapeutic targeting in IBD.

Accordingly, this narrative review briefly introduces the intestinal localization and homeostatic functions of ILC subsets, with a primary focus on the evidence, molecular mechanisms, and functional consequences of ILC plasticity in IBD-associated immune barrier remodeling. Evidence obtained from human IBD tissues and relevant experimental colitis models is prioritized, whereas findings from other mucosal tissues are included only when they provide mechanistic insight into the plastic potential of ILCs or highlight unresolved questions relevant to intestinal inflammation. It further discusses the current limitations of the field and the therapeutic potential of targeting disease- and context-specific ILC fate transitions to restore intestinal immune homeostasis. Throughout this review, intestinal immune barrier remodeling is therefore considered as an integrative framework encompassing epithelial integrity and repair, immune-cell composition, cytokine balance, and tissue-remodeling responses, with particular emphasis on how context-dependent ILC-state transitions may influence these interconnected components.

## 2 Intestinal ILC subsets relevant to IBD and plasticity

All ILC populations, including ILC1s, ILC2s, ILC3s, natural killer (NK) cells, and lymphoid tissue inducer (LTi) cells, originate from common lymphoid progenitors in the bone marrow. One developmental branch forms NK-cell progenitors and ultimately conventional NK cells under the regulation of Eomes and T-bet, whereas another generates common helper innate lymphoid progenitors. These progenitors further give rise to PLZF^+^ innate lymphoid cell progenitors (ILCPs) and PLZF^-^ LTi progenitors. PLZF^+^ ILCPs mature into ILC1s, ILC2s, and conventional ILC3s under transcriptional programs involving T-bet, GATA3, and RORγt, respectively, whereas PLZF^-^ progenitors specifically generate LTi cells (16, 17).

**Figure 1:**
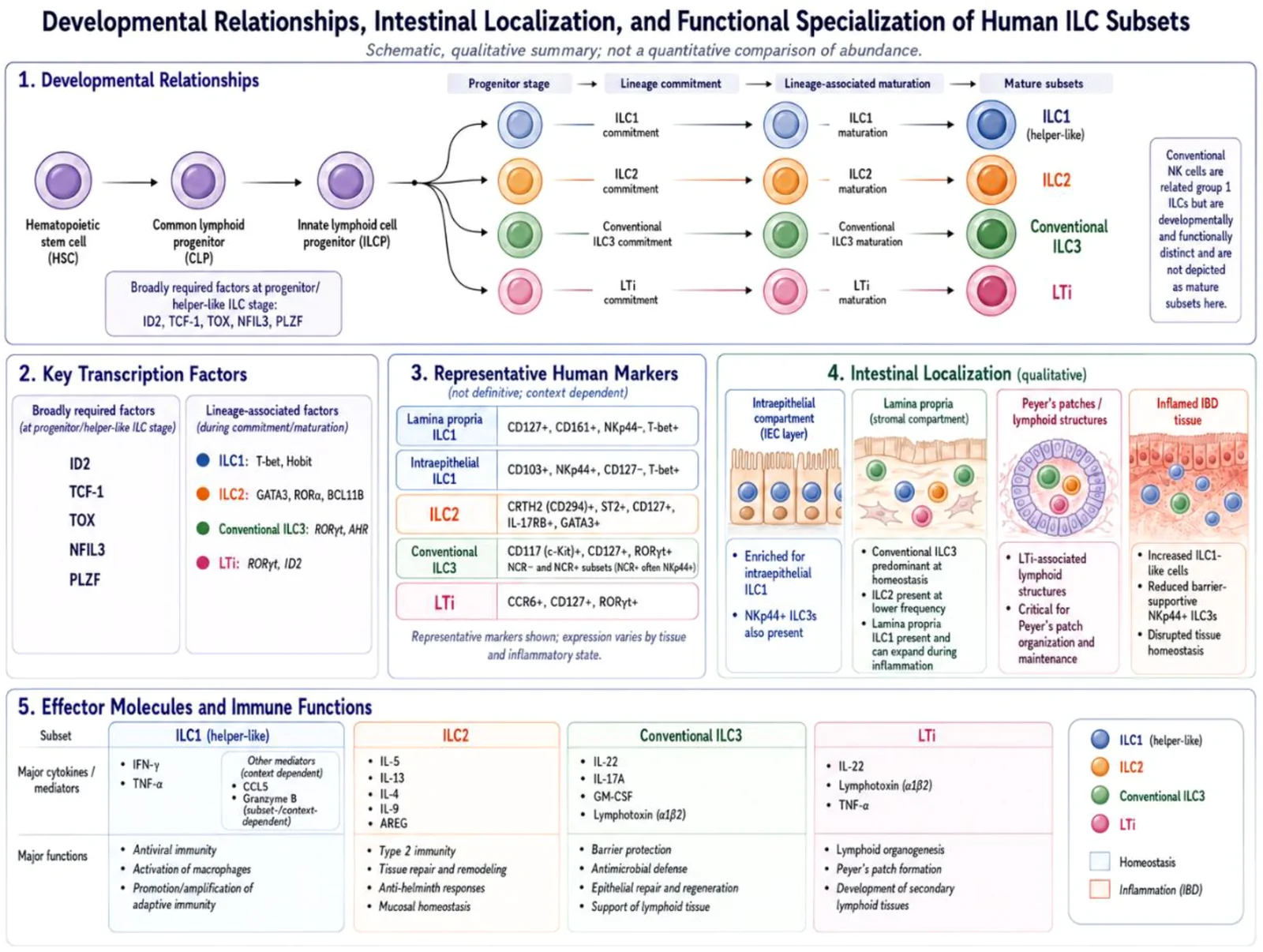
Developmental relationships, intestinal localization, and functional specialization of human ILC subsets.

According to their transcription-factor expression and cytokine profiles, ILCs are classified into group 1 ILCs, comprising NK cells and ILC1s; group 2 ILCs, comprising ILC2s; and group 3 ILCs, comprising conventional ILC3s and LTi cells (14) (**Figure 1**). Because conventional NK cells have developmental and cytotoxic programs distinct from helper-like ILCs, the following sections focus mainly on intestinal ILC1s, ILC2s, and group 3 ILC populations (18).

### 2.1 Intestinal ILC1s: localization and inflammatory functions

Group 1 ILCs comprise conventional NK cells and ILC1s. Both express T-bet and participate in type 1 immune responses, but their principal effector mechanisms differ (18–22). Conventional NK cells are abundant in the circulation and peripheral immune organs and exert strong cytotoxic activity through perforin and granzymes (18, 23, 24), whereas ILC1s are predominantly tissue-resident cells that rapidly produce IFN-γ and TNF-α to regulate local immune responses (21, 25).

In the intestine, ILC1s can be broadly divided into lamina propria ILC1s (LP-ILC1s) and intraepithelial ILC1s (ieILC1s) according to their anatomical localization and phenotype (21). Human LP-ILC1s are generally CD127^+^CD161^+^NKp44^-^T-bet^+^Eomes^-^ cells and primarily produce IFN-γ and TNF-α, thereby promoting macrophage and dendritic-cell activation; they exhibit little or no perforin- or granzyme-mediated cytotoxicity (21, 22, 26). In contrast, ieILC1s are mainly located in the epithelial compartment and commonly display a CD103^+^NKp44^+^CD127^-^T-bet^+^Eomes^+^ phenotype. These cells have limited cytotoxic potential and rapidly respond to IL-12, IL-15, and IL-18 by producing IFN-γ (21, 22, 26, 27).

ILC1s should not be regarded as the predominant source of IFN-γ during intestinal inflammation. CD4^+^ T cells, CD8^+^ T cells, conventional NK cells, γδ T cells, and natural killer T cells can also contribute substantially to IFN-γ production. ILC1s are more appropriately considered an important early, tissue-resident innate source because they can respond rapidly without prior antigen-specific activation (28–30).

In both newly diagnosed and established CD, the proportion of ILC1s is increased in the intestinal mucosa, particularly in inflamed small-intestinal tissue, whereas ILC frequencies in non-inflamed areas may remain comparable to those in non-IBD controls (20). Activated ILC1s in the CD microenvironment produce IFN-γ and other inflammatory mediators that may impair epithelial-barrier integrity, increase intestinal permeability, and promote mucosal inflammation (20, 21, 31, 32).

In anti-CD40-induced colitis in Rag1^-^/^-^ mice, ieILC1s rapidly produced IFN-γ during early inflammation. Depletion of NK1.1^+^ cells reduced inflammatory infiltration and epithelial injury; however, anti-NK1.1 treatment depletes both ILC1s and conventional NK cells and therefore does not represent an ILC1-specific knockout model (21). These findings support the involvement of NK1.1^+^ group 1 innate lymphocytes but do not allow the protective effect to be attributed exclusively to ILC1 depletion.

In established UC, LP-ILC1 frequencies are also increased in inflamed mucosa, whereas ieILC1 and NK-cell populations are reduced relative to healthy controls (26). In experimental colitis, IFN-γ- and TNF-α-associated inflammatory signaling can reduce epithelial tight-junction proteins, impair barrier integrity, and aggravate mucosal damage (21, 27, 30, 33, 34). ILC1-derived IFN-γ may additionally promote pro-inflammatory macrophage polarization (27). Conversely, macrophage-derived CCL1 can signal through CCR8 to regulate IFN-γ-producing intestinal ILCs and induce mucosal-protective genes such as IDO1, indicating that ILC1 functions in UC are context dependent (35).

### 2.2 Intestinal ILC2s: type 2 immunity and epithelial repair

ILC2s are helper-like ILCs characterized principally by GATA3 expression and are distributed across barrier tissues and lymphoid organs (36–40). Based on their phenotype, tissue residency, and responsiveness to epithelial-derived cytokines, ILC2s are commonly divided into natural ILC2s (nILC2s; IL-17RB^-^ST2^+^) and inflammatory ILC2s (iILC2s; IL-17RB^+^ST2^-^) (36, 41, 42).

nILC2s are tissue-resident cells that respond primarily to IL-33 and produce IL-5 and IL-13. They contribute to type 2 immunity, tissue repair, anti-helminth defense, and maintenance of mucosal-barrier homeostasis (43–51). Under steady state, intestinal ILC2 populations consist predominantly of nILC2s and are relatively sparse in the colon, with greater enrichment in the small-intestinal lamina propria. Tuft-cell-derived IL-25 supports their survival and basal activity (26, 44). During helminth infection, ILC2-derived IL-13 promotes goblet-cell expansion and mucus production, while amphiregulin supports epithelial repair and smooth-muscle responses facilitate parasite expulsion (44). In murine intestinal injury, IL-33-activated gut-associated ILC2s upregulated amphiregulin, and the ILC2–amphiregulin–EGFR pathway promoted epithelial-barrier restoration and reduced intestinal disease severity (52).

By contrast, iILC2s emerge mainly following strong IL-25 stimulation or helminth-associated inflammation and can migrate through the circulation to inflammatory tissues (44). Compared with tissue-resident nILC2s, iILC2s display greater migratory capacity and broader context-dependent cytokine potential. However, because they have been characterized primarily following experimental IL-25 administration or parasite infection, their direct relevance to human intestinal IBD remains uncertain.

In IBD, intestinal ILC2s may exert both reparative and disease-promoting effects. ILC2-derived mediators can support epithelial proliferation, goblet-cell differentiation, mucus-barrier restoration, and tissue repair, whereas persistent activation may contribute to excessive inflammation and fibrosis (53). IL-33-activated ILC2s produce IL-13 and promote goblet-cell differentiation and intestinal homeostasis (54). In DSS-induced colitis, transfer of purified ILC2s alleviated acute disease and was accompanied by increased IL-5 and IL-13 levels and enhanced M2-like macrophage polarization (55). Epithelial-derived IL-25, IL-33, and thymic stromal lymphopoietin may further activate intestinal ILC2s and contribute to barrier maintenance and tissue repair (56).

Human mucosal studies further suggest that alterations in ILC2 abundance depend on disease subtype and stage. ILC2 frequencies are increased in newly diagnosed UC but not consistently in newly diagnosed CD, whereas elevated ILC2 frequencies have been observed in both established CD and established UC. Nevertheless, changes in ILC2 abundance do not themselves demonstrate lineage conversion. Direct evidence of directional ILC2 plasticity in intestinal tissues from patients with CD or UC remains limited; therefore, context-dependent ILC2 plasticity is discussed separately in Section 3.

### 2.3 Intestinal group 3 ILCs: mucosal defense and lymphoid-tissue organizations

Group 3 ILCs comprise CCR6^+^ lymphoid tissue inducer (LTi) cells and conventional CCR6^-^ ILC3s. Both populations depend on RORγt for their lineage identity, while AhR signaling contributes to the development and maintenance of conventional ILC3s (29, 57, 58). LTi cells are developmentally specialized group 3 ILCs with a critical role in embryonic lymphoid-organ formation, whereas conventional CCR6^-^ ILC3s can be further classified as NCR^+^ or NCR^-^ subsets according to their expression of natural cytotoxicity receptors.

Under steady state, group 3 ILCs support lymphoid-tissue organization, intestinal mucosal defense, and epithelial-barrier maintenance. LTi cells contribute primarily to the formation and organization of intestinal lymphoid structures, whereas conventional ILC3 subsets exert distinct effector functions in the intestinal mucosa. During embryonic development, LTi cells are essential for secondary lymphoid-organ formation (59). In experimental intestinal inflammation, IL-23-responsive RORγt^+^ innate lymphoid cells accumulated in the inflamed colon and produced IL-17 and IFN-γ, thereby contributing directly to innate intestinal pathology (60). By contrast, NCR^+^ ILC3s predominantly produce IL-22, activate STAT3 signaling in intestinal epithelial cells, and support epithelial regeneration (61). IL-22 also induces antimicrobial peptides such as RegIIIγ and RegIIIβ (61). In IBD, IL-23/IL-17 pathway activation may enhance inflammatory ILC3 responses and exacerbate mucosal injury (62), while loss of IL-22-producing ILC3s may weaken epithelial repair. These context-dependent effects indicate that ILC3s should not be classified simply as either protective or pathogenic. Experimental evidence further supports a context-dependent contribution of IL-17-producing IL-7R^+^ ILCs to intestinal inflammation. In a T-bet-dependent mouse model of innate immune-mediated colitis, neutralization of IL-17A markedly ameliorated intestinal inflammation and resulted in complete resolution in most treated mice (63). However, these findings were obtained from an experimental colitis model and should not be directly interpreted as evidence that anti-IL-17A treatment completely resolves human UC or that all IL-17-producing ILC3s are pathogenic in human IBD.

Human mucosal studies have revealed disease- and stage-specific alterations in intestinal ILC composition. Compared with non-IBD controls, IBD mucosal samples showed a shift toward increased frequencies of ILC1s and NKp44^-^ ILC3s and reduced frequencies of NKp44^+^ ILC3s. ILC2s, which were nearly undetectable in most non-IBD mucosal samples, were also detected more frequently in patients with IBD. NKp44^+^ ILC3 frequencies were reduced in inflamed mucosal samples from both CD and UC, irrespective of whether the disease was newly diagnosed or established. In contrast, ILC1 frequencies were increased in newly diagnosed CD but not in newly diagnosed UC, whereas ILC2 frequencies were increased in newly diagnosed UC but not in newly diagnosed CD. In established CD and UC, both ILC1 and ILC2 frequencies were elevated. Increased NKp44^-^ ILC3 frequencies were also detected in newly diagnosed CD and established UC. Moreover, ieILC1 frequencies were lower in newly diagnosed UC but increased in newly diagnosed CD, whereas established UC was associated with reduced frequencies of both ieILC1s and conventional NK cells (26). Consistent with these findings, earlier studies of inflamed CD mucosa demonstrated an accumulation of IFN-γ-producing ILC1s, accompanied by a reduction in NKp44^+^ ILC3s and impaired IL-22 production, particularly in the terminal ileum (20, 64).

Additional analysis of the terminal ileum in patients with CD showed that NKp44^+^ ILC3s were the predominant ILC population in unaffected tissue, accounting for approximately 60% of total ILCs. These cells produced high levels of IL-22 but little or no IL-17A or IFN-γ. In inflamed terminal ileal tissue, however, the frequency of NKp44^+^ ILC3s and their IL-22 production were reduced, while the remaining NKp44^+^ ILC3s acquired limited IFN-γ-producing capacity. At the same time, NKp44^-^CD117^-^ ILC1s and IFN-γ-producing ILC1 populations increased, whereas ILC1s from both unaffected and inflamed tissues did not produce IL-22 (64). Consistent with this shift toward group 1 ILC-associated inflammation, a subsequent single-cell and flow-cytometric study identified a marked expansion of granulysin-expressing CD127^+^CD94^+^CD117^-^ group 1 ILCs in inflamed intestinal tissues from patients with active CD (65). More recent large-scale single-cell atlases further demonstrate substantial inflammation- and intestinal-segment-dependent remodeling of immune-cell composition in CD, although they do not resolve the same NKp44-defined ILC subsets (66).

Collectively, flow-cytometric and single-cell studies indicate that active CD is generally associated with increased intestinal ILC1 frequencies and reduced ILC3 frequencies, whereas changes in ILC2 populations are more heterogeneous and depend on tissue location, disease subtype, and disease stage (20). Large-scale single-cell analysis further demonstrate that immune-cell composition in CD varies substantially according to intestinal segment, tissue compartment, and inflammatory status (67). Nevertheless, altered subset frequencies cannot be explained exclusively by lineage conversion, because recruitment, migration, local proliferation, survival, and apoptosis may produce similar changes. Changes in abundance should therefore not be considered direct evidence of plasticity. Instead, evidence for dynamic and potentially reversible fate transitions is provided by studies identifying cytokine-dependent bidirectional conversion and transcriptionally intermediate ILC3–ILC1 states (68). The following section discusses the principal patterns of ILC plasticity and evaluates the strength of the evidence linking them to intestinal inflammation. .

The figure provides a qualitative schematic summary of the developmental relationships among conventional NK cells, helper-like ILC1s, ILC2s, conventional ILC3s, and LTi cells, together with representative lineage-associated transcription factors, phenotypic markers, intestinal localization patterns, and major effector functions. The developmental sequence does not imply an irreversible terminal-differentiation hierarchy, and the tissue-distribution component is not intended as a quantitative comparison of subset abundance. Helper-like ILC1s are primarily associated with rapid IFN-γ and TNF-α production, whereas conventional NK cells additionally express Eomes and exert prominent perforin- and granzyme-dependent cytotoxicity. ILCP, innate lymphoid cell progenitor. This figure is an original schematic illustration created by the authors based on the cited literature and is not reproduced or directly adapted from a previously published figure.

## 3 ILC plasticity in IBD-associated intestinal immune barrier remodeling

ILC subsets share developmental origins and portions of their transcriptional regulatory networks, providing a molecular basis for fate transitions. Although each subset has relatively stable lineage-associated characteristics, the regulatory networks of mature ILCs retain sufficient accessibility to adjust phenotype and function in response to local tissue signals (14, 69). ILC plasticity refers to reversible remodeling of the phenotype, function, and transcriptional program of mature ILCs in response to cytokines, tissue-derived signals, metabolic reprogramming, and epigenetic regulation, resulting in the acquisition of selected characteristics of another ILC subset (14, 69). This process differs from differentiation, which occurs during the development of progenitors into mature cells and generally involves stable silencing of alternative lineage programs. In mature ILCs, environmental signals can dynamically alter lineage-defining transcription factors, and removal of the relevant stimulus may permit recovery of the original phenotype (14, 69–72).

ILC plasticity contributes to tissue homeostasis and chronic inflammatory responses and may participate directly in intestinal immune barrier remodeling (21). The remodeling patterns of CD and UC differ substantially. CD is characterized by segmental transmural inflammation, dysregulated type 1 and type 17 responses, lymphoid-tissue expansion, stromal fibrosis, and a risk of intestinal stenosis; directional conversion of ILC3s toward ILC1-like states represents an important innate immune component of this pathological remodeling (14, 22, 73, 74). In contrast, UC inflammation is largely confined to the colonic mucosa and submucosa and is characterized by continuous superficial ulceration, goblet-cell loss, mucus-layer disruption, and altered type 2 immunity. A defined directional ILC transition has not yet been demonstrated in UC (75, 76).

Within these disease-specific microenvironments, epithelial injury, microbial dysbiosis, and sustained production of inflammatory cytokines alter both the abundance and functional states of intestinal ILC subsets. IL-12 and IL-15 can favor IFN-γ-producing ILC1-like states, whereas loss or functional impairment of IL-22-producing ILC3 populations may weaken epithelial regeneration and antimicrobial defense (14, 69, 77, 78). ILC-derived mediators may, in turn, shape the surrounding immune compartment: IFN-γ can promote inflammatory macrophage and dendritic-cell activation and impair epithelial-barrier function; IL-17 can enhance chemokine production and neutrophil recruitment; and IL-22 acts directly on intestinal epithelial cells to support repair and antimicrobial defense (27, 77, 78). Cytokines produced by dendritic cells and macrophages, including IL-12, IL-23, IL-1β, and CCL1, can reciprocally regulate ILC phenotype and function, thereby establishing bidirectional interactions between ILCs and the inflamed intestinal microenvironment (14, 15, 51). Direct regulation of adaptive immunity by intestinal ILC3s has also been demonstrated through MHC class II-dependent interactions that limit commensal bacteria-specific CD4^+^ T-cell responses. Notably, MHC class II expression on colonic ILC3s was reduced in pediatric IBD, suggesting that this regulatory pathway may be disrupted during intestinal inflammation (79). However, its quantitative contribution across adult CD and UC remains incompletely defined.

Although NK cells and ILC1s both belong to group 1 ILCs, NK cells primarily mediate cytotoxicity and differ from helper-like ILCs in their developmental programs and biological functions (18, 21). Because NK cells are relatively infrequent in the intestine and direct evidence linking NK-cell plasticity to IBD remains limited, the following discussion focuses on helper-like ILC1s, ILC2s, and ILC3s (26).

### 3.1 ILC3-to-ILC1 plasticity in crohn’s disease

ILC1s and ILC3s exhibit bidirectional plasticity. NCR^+^RORγt^+^ ILC3s maintain RORγt expression in response to IL-7, whereas IL-12 and IL-15 promote RORγt downregulation. Loss of RORγt can drive ILC3s toward a type 1 immune phenotype with increased IFN-γ production and inflammatory activity. Intermediate ILC3-ILC1 states have been identified in human tonsils and ileal mucosa (68). Mechanistically, T-bet suppresses the ILC3-associated transcriptional program and promotes the acquisition of ILC1-like features (29, 80). During this transition, IL-23 and commensal-derived signals contribute to T-bet induction, whereas IL-12 strongly promotes IFN-γ production and functional maturation of T-bet-directed NKp46^+^ ILC3s (29). ILC3s transferred into the spleens of humanized mice produce more IFN-γ and less IL-22, further illustrating the influence of tissue context on ILC identity (68).

Reversible ILC3-to-ILC1 conversion has been reported in CD (14, 81). Increased CD127^+^ ILC1s and reduced ILC3s in the intestinal lamina propria are consistent with this transition (14). During inflammation, IL-12 produced by CD14^+^ dendritic cells promote conversion toward ILC1-like cells, whereas IL-23 and IL-1β from CD14^-^ dendritic cells can support re-differentiation toward ILC3s after inflammation resolves; retinoic acid further enhances this reverse transition (14). RORγt is required to maintain ILC3 identity, and its loss increases T-bet expression and favors ILC1-like reprogramming (80). In experimental colitis, wogonin activates the AhR/IL-22 pathway, increases the RORγt/T-bet ratio, limits ILC3-to-ILC1 conversion, and alleviates DSS-induced disease (82, 83). Functionally, this transition may contribute to intestinal immune barrier remodeling by replacing an IL-22-dominant, epithelial-supportive ILC3 program with an IFN-γ-dominant inflammatory program, thereby coupling altered ILC identity to impaired epithelial repair and enhanced mucosal inflammation.

### 3.2 Context-dependent ILC2 plasticity

ILC2s can acquire ILC1-like or ILC3-like characteristics under selected inflammatory conditions, although these transitions have not been established as major pathogenic mechanisms in intestinal IBD. Under inflammatory stimulation, some ILC2s may lose part of their original GATA3-dependent phenotype and acquire features associated with another ILC lineage; these phenotypically reprogramed populations are sometimes referred to as ex-ILC2s. IL-25-responsive KLRG1^hi inflammatory ILC2s provide experimental evidence of this broader plastic potential. Compared with lung nILC2s and small-intestinal lamina propria ILC3s, mouse iILC2s expressed high levels of GATA3 together with intermediate levels of RORγt. Consistent with this mixed transcription-factor profile, a small fraction of freshly isolated IL-13-producing iILC2s also produced IL-17 after stimulation. These cells were capable of generating nILC2-like or ILC3-like progeny, indicating that iILC2s possess context-dependent plasticity or multipotentiality rather than a completely fixed effector program (43). However, these findings were obtained primarily in IL-25-driven mouse models and should not be directly extrapolated to human intestinal IBD.

Notably, IL-13^+^IFN-γ^+^ ILC2-like cells have been identified in intestinal samples from patients with Crohn’s disease (CD), and IL-12 stimulation can induce IFN-γ expression in human IL-13^+^ ILC2-derived populations, supporting the occurrence of type 1-skewed ILC2 phenotypes in the diseased intestine (69). However, the lineage stability, abundance, and pathogenic contribution of these cells in CD remain incompletely defined. IL-1β pretreatment increases IL-12 responsiveness and promotes T-bet expression, generating an ILC1-like phenotype through coordinated regulation of T-bet, IL-5, IL-13, IL-12Rβ2, and IFN-γ-associated programs (84). In severe chronic obstructive pulmonary disease, IL-12 promotes ILC2-to-ILC1-like phenotypic drift, whereas IL-4 helps preserve ILC2 identity (85). Influenza virus and cigarette-smoke exposure similarly reduce GATA3 and increase IL-12Rβ2, IL-18Rα, T-bet, and IFN-γ in mouse pulmonary ILC2s (86). Comparable IL-12-driven changes have also been observed in human peripheral-blood ILC2s (69, 85). These findings indicate that IL-1β and IL-12 can promote type 1-like reprogramming of ILC2s across inflammatory settings, but the causal importance of this transition in intestinal IBD remains uncertain.

ILC2 plasticity may also involve the acquisition of ILC3-like characteristics. In psoriatic skin, c-Kit^+^CCR6^+^ ILC2-like populations can express RORγt and produce IL-17 after IL-1β and IL-23 stimulation. Increased RORγt and reduced GATA3 are key features of this transition; RORγt inhibition reduces IL-17 production, whereas high GATA3 expression suppresses the ILC3-like program (87). Together, these studies demonstrate the broader plastic potential of ILC2s in inflammatory tissues but do not establish that comparable ILC2-to-ILC3-like transitions occur or contribute directly to disease in human intestinal IBD. Accordingly, ILC2 plasticity could influence intestinal immune barrier remodeling by altering the balance between reparative effector programs and IFN-γ- or IL-17-associated inflammatory states, but this proposed link remains insufficiently demonstrated in human intestinal IBD.

### 3.3 Epigenetic and metabolic reprogramming

In addition to cytokine-driven regulation, ILC plasticity is shaped by epigenetic and metabolic reprogramming (88). Genome-wide profiling of small-intestinal ILC subsets has identified subset-specific active promoter and enhancer landscapes marked by H3K4me3 and H3K27ac, respectively. Maintenance of H3K4me3 also contributes to intestinal ILC3 homeostasis and effector-gene expression (89, 90). Lineage-associated transcription factors cooperate with chromatin regulators to establish subset-specific epigenetic programs (91). Direct studies provide examples of epigenetic regulators acting predominantly during lineage establishment or subset differentiation. Brg1 promotes the differentiation of NKp46^+^ ILC3s by regulating the T-bet-associated program and restrains GM-CSF-associated intestinal inflammation, whereas G9a supports ILC2 development by repressing the ILC3-associated gene program (92, 93). These findings primarily concern ILC lineage establishment or subset differentiation rather than direct plasticity of mature ILCs.

By contrast, plasticity in mature ILCs may involve dynamic and context-dependent changes in chromatin accessibility and histone modification during inflammation. In mature CCR6^-^ ILC3s, c-Maf maintains the RORγt-associated type 3 program and restrains T-bet-driven acquisition of type 1 features by regulating transcriptional and chromatin-accessibility states (94). More recent evidence indicates that the H3K27 demethylases UTX and JMJD3 regulate intestinal ILC3 subset plasticity by removing H3K27me3, maintaining accessibility of a *Tcf7*-associated enhancer, and controlling the balance among double-negative, NKp46^+^, and CCR6^+^ ILC3 states (95). Together, these studies provide direct evidence that dynamic chromatin regulation contributes to mature ILC3-state remodeling.

Metabolic requirements differ among ILC subsets and activation contexts and can influence their homeostasis and effector functions. Activated ILC3s engage an mTORC1–HIF-1α-dependent glycolytic program to support proliferation and effector-cytokine production (96). Hypoxic conditions can also enhance RORγt-associated ILC3 responses through HIF-1α-dependent mechanisms, although some hypoxia-induced effects may occur independently of mTORC1, indicating that HIF-1α regulation is context dependent (97). LKB1 also supports intestinal ILC3 homeostasis by maintaining mitochondrial function, cellular survival, and IL-22 production. ILC3-specific LKB1 deficiency causes mitochondrial bioenergetic dysfunction and lipid accumulation, accompanied by reduced ILC3 abundance and impaired effector function (98). Mitochondrial transcription factor A (TFAM) also contributes to the maintenance of small-intestinal RORγt^+^ lymphocyte populations and intestinal metabolic homeostasis. In Rorc-Cre-driven Tfam-deficient mice, intestinal ILC3 abundance was reduced and small-intestinal homeostasis was altered; however, because the model also affects other RORγt^+^ lymphocytes, these findings cannot be attributed exclusively to an ILC3-intrinsic function of TFAM (99). Metabolic requirements also differ according to ILC subset and activation state. Steady-state human peripheral-blood ILC2s display substantial reliance on oxidative phosphorylation, whereas glycolysis is preferentially engaged to support selected effector functions after activation (100). In the intestine, ILC2s can additionally rely on fatty-acid metabolism to sustain proliferation, type 2 cytokine responses, and barrier-protective functions during helminth infection and nutritional stress (101). Together, these findings indicate that ILC metabolic programs are dependent on cell subset, tissue context, nutritional status, and activation state, rather than being uniformly divided into inflammatory glycolytic and homeostatic oxidative programs. However, most of these studies primarily address ILC maintenance, bioenergetics, or effector function rather than direct lineage conversion; whether metabolic reprogramming causally drives ILC plasticity in human IBD therefore remains unresolved. Taken together, ILC plasticity may provide a mechanism through which changes in the local inflammatory microenvironment are translated into altered barrier-regulatory effector programs. In CD, ILC3-to-ILC1-like remodeling may simultaneously reduce IL-22-dependent epithelial support and antimicrobial defense while increasing IFN-γ-associated mucosal inflammation. Conversely, preservation or restoration of barrier-supportive ILC3 states may favor epithelial repair and immune homeostasis. For ILC2s, comparable effects on repair, inflammation, and fibrosis are biologically plausible but remain less directly established in human intestinal IBD. Thus, the consequences of ILC plasticity for intestinal immune barrier remodeling are likely disease-, tissue-, and state-dependent rather than uniform across IBD.

**Figure 2:**
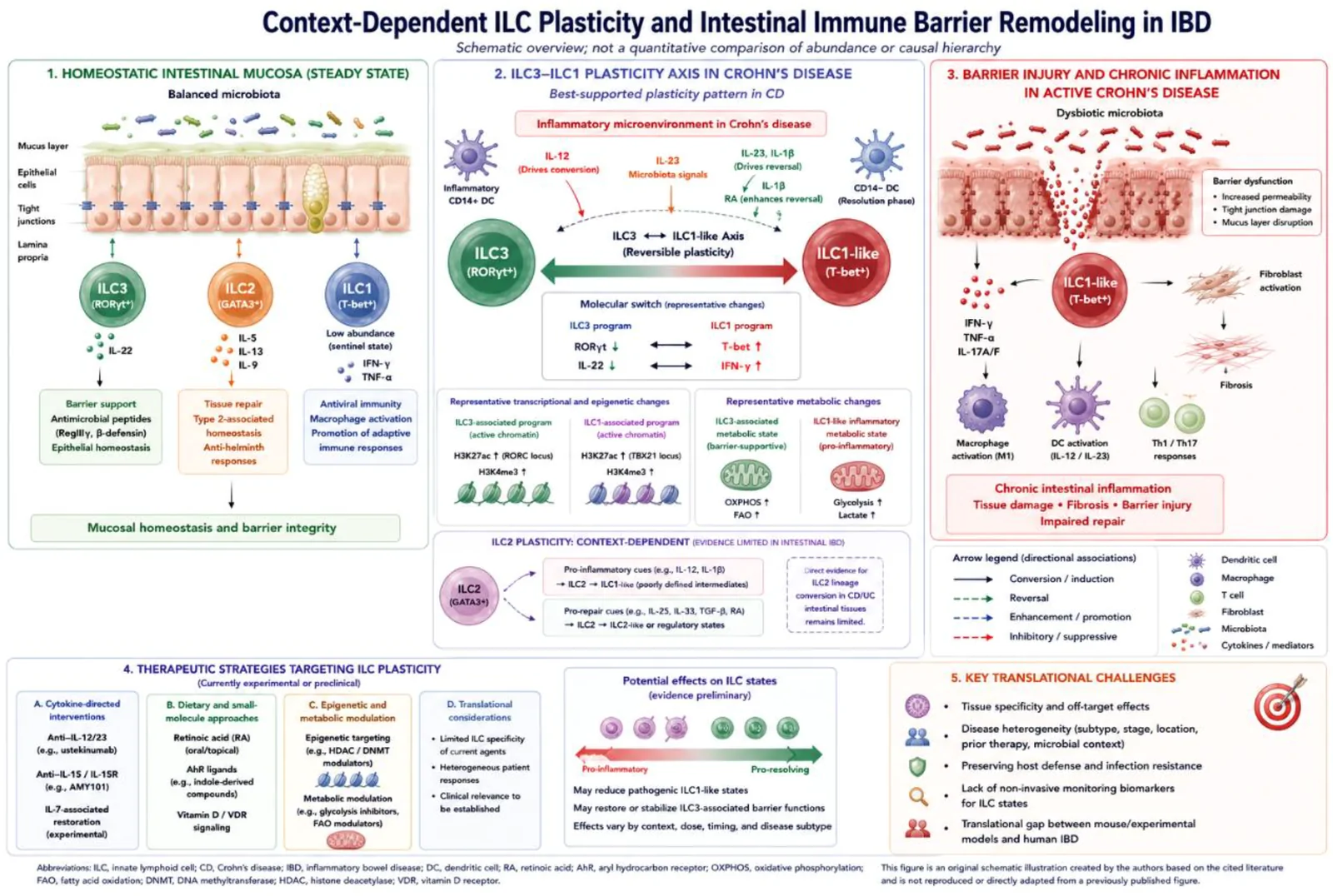
Context-dependent ILC plasticity in intestinal immune barrier remodeling.

The context-dependent relationships among homeostatic ILC functions, inflammatory ILC-state remodeling, epithelial-barrier injury, and potential therapeutic interventions are summarized schematically in **Figure 2**. Representative evidence for ILC plasticity across intestinal and extraintestinal disease contexts is summarized in **Table 1**.

**Table 1:** Representative evidence for ILC plasticity across tissues and disease contexts.

Disease or experimental context	Tissue/site	Key inducing signals or microenvironmental factors	Relevance to intestinal IBD	Species	Observed plasticity	Refs
Experimental intestinal inflammation/colitis	Intestinal lamina propria	IL-7 and intestinal microbiota maintain RORγt expression, whereas IL-12 and IL-15 promote RORγt loss and acquisition of an IFN-γ-producing phenotype	Direct preclinical evidence for intestinal ILC3-to-ILC1-like conversion	Mouse	NCR^+^RORγt^+^ ILC3s → IFN-γ-producing ILC1-like cells	(102)
Crohn’s disease	Intestinal lamina propria and terminal ileum	IL-12 promotes ILC3-to-ILC1 conversion; IL-23, IL-1β, IL-2, and retinoic acid can promote reacquisition of ILC3 characteristics	Direct human evidence relevant to CD	Human	CD127^+^ ILC3s ↔ CD127^+^ ILC1-like cells	(14, 20, 82)
Severe COPD and CRSwNP	Lung and upper-airway mucosa	IL-1β activates ILC2s, IL-12 induces T-bet and IFN-γ, and IL-4 preserves or restores ILC2 identity	Extraintestinal mechanistic evidence for cytokine-dependent ILC2 plasticity	Human	ILC2s → IFN-γ-producing ILC1-like cells	(85)
Influenza- and cigarette-smoke-associated COPD-like inflammation	Lung	Viral infection and cigarette-smoke exposure induce loss of the ILC2 program and acquisition of type 1 features	Extraintestinal preclinical evidence	Mouse	ILC2s → ILC1-like cells	(86)
Psoriasis	Skin and peripheral blood	IL-1β and IL-23 induce RORγt and IL-17, accompanied by reduced GATA3 expression	Extraintestinal evidence illustrating ILC2-to-ILC3-like potential	Human	c-Kit^+^CCR6^+^ ILC2s → IL-17-producing ILC3-like cells	(87)
Cystic fibrosis-associated nasal inflammation	Nasal mucosa	Epithelial-derived IL-1β, IL-23, and TGF-β induce RORγt and IL-17 production; IL-4 and vitamin D_3_ inhibit the transition	Extraintestinal mucosal evidence; intestinal relevance remains unconfirmed	Human	ILC2s → IL-17-producing ILCs	(103)
Silicosis	Lung	Mechanics-activated fibroblasts promote conversion through Notch3–IL-18-associated signaling	Extraintestinal evidence linking stromal remodeling to ILC plasticity	Mouse	ILC2s → ILC1-like cells	(104)
Fibrosarcoma	Tumor microenvironment	Tumor-derived TGF-β signaling promotes loss of NK-cell cytotoxic identity and acquisition of ILC1-like features	Demonstrates that tissue-derived signals can remodel mature group 1 innate lymphocytes; indirect relevance to IBD	Mouse	Conventional NK cells → ILC1-like cells	(105)
Squamous cell carcinoma	Lung tumor tissue	Tumor-cell-derived IL-23 promotes acquisition of RORγt- and IL-17-associated ILC3-like features	Supports cytokine-driven ILC1-to-ILC3-like plasticity outside the intestine	Human	ILC1s → ILC3-like cells	(106)
Colon cancer	Colorectal tumor microenvironment	Tumor-associated inflammatory signals disrupt ILC3 identity and barrier-supportive functions	Anatomically relevant to the intestine, but represents cancer-associated rather than IBD-associated remodeling	Human	ILC3s → ILC1-like or dysfunctional inflammatory states	(107)
Melanoma	Tumor tissue	IL-12 induces IFN-γ production and antitumor activity	Shows functional reprogramming of group 3 ILCs; evidence for complete lineage conversion is limited	Mouse	NKp46^+^RORγt^+^ ILC3/LTi-like cells acquire type 1 effector functions	(108)
Hepatocellular carcinoma	Liver tumor microenvironment	Tumor-derived cytokines and local tissue signals reshape the ILC transcriptional landscape	Demonstrates tissue-dependent intermediate states, but is indirectly related to intestinal IBD	Human	NK-like cells acquire ILC1- and ILC3-associated states	(109)
NAFLD/obesity-associated liver disease	Liver	Obesity-associated hepatic signals, including TGF-β-related pathways	Supports metabolic-microenvironmental regulation of group 1 ILC identity	Mouse	Conventional NK cells → less cytotoxic ILC1-like cells	(110)

CD, Crohn’s disease; COPD, chronic obstructive pulmonary disease; CRSwNP, chronic rhinosinusitis with nasal polyps; IBD, inflammatory bowel disease; ILC, innate lymphoid cell; LTi, lymphoid tissue inducer; NAFLD, non-alcoholic fatty liver disease; NCR, natural cytotoxicity receptor.

Under homeostatic conditions, ILC2s and ILC3s contribute to epithelial repair, antimicrobial defense, and mucosal barrier maintenance. In Crohn’s disease, inflammatory cytokines and microbial signals may promote ILC3-to-ILC1-like remodeling, characterized by reduced RORγt and IL-22 expression and increased T-bet and IFN-γ production. This process is associated with transcriptional, epigenetic, and metabolic changes and may be partially reversible during inflammation resolution. Cytokine-directed therapies, retinoic acid, and AhR-related interventions may indirectly influence these ILC states, although their ILC specificity and clinical relevance remain to be established. Arrows indicate reported or proposed directional associations and do not imply equivalent evidence strength or direct causality. The terms “Type 1 immunity” and “Type 2 immunity” denote broad functional immune-response patterns rather than distinct ILC lineages. This figure is an original illustration created by the authors based on the cited literature and is not reproduced from a previously published source.

## 4 Therapeutic implications: targeting ILC plasticity

In selected inflammatory contexts, otherwise homeostatic ILC subsets may acquire disease-associated phenotypes through plasticity. Because these state transitions may alter epithelial repair, antimicrobial defense, cytokine balance, and interactions with other immune cells, modulating them may represent a potential strategy for restoring intestinal immune barrier homeostasis in IBD. Modulating these context-dependent transitions may therefore represent a potential therapeutic strategy for IBD. However, current evidence is derived mainly from mechanistic studies and experimental models, and most available interventions affect cytokine, transcriptional, or metabolic pathways shared by multiple immune-cell populations rather than targeting ILCs selectively. The following sections distinguish clinically established therapies that may indirectly influence ILC states from preclinical interventions intended to stabilize barrier-supportive ILC programs.

### 4.1 Cytokine regulation and modulation of ILC fate

Cytokines involved in ILC3–ILC1 plasticity are pharmacologically accessible, but their effects on ILC fate should be interpreted as one component of broader immunomodulation. IL-12 and IL-15 favor inflammatory ILC1-like programs, whereas IL-7 can support RORγt expression and IL-22-associated ILC3 functions (14, 102). Because these pathways also regulate T cells, NK cells, and other lymphocyte populations, the therapeutic effects of cytokine-directed interventions cannot currently be attributed specifically to modulation of ILC plasticity.

Among clinically available agents, ustekinumab targets the shared p40 subunit of IL-12 and IL-23 and has demonstrated sustained clinical efficacy in patients with Crohn’s disease (111, 112). By suppressing IL-12/IL-23 signaling, ustekinumab may reduce the inflammatory conditions that favor ILC3-to-ILC1-like remodeling. However, direct evidence that inhibition of this transition is a major mechanism underlying its clinical efficacy remains lacking. Its effects on ILC states should therefore be regarded as a plausible secondary mechanism rather than an established therapeutic mode of action.

IL-15-directed interventions remain at an earlier stage of development. In DSS-induced colitis, IL-15 neutralization reduced inflammatory-cell infiltration, mucosal injury, and the expression of inflammatory mediators (113). Selective blockade of IL-15Rα has also been reported to reduce IL-15-dependent inflammatory signaling in preclinical models (114). These findings support further investigation of the IL-15 pathway, although its broad role in NK-cell and T-cell homeostasis raises concerns regarding infection risk and immune suppression.

IL-7 signaling can stabilize RORγt expression in selected intestinal ILC3 states. However, recombinant IL-7 has not been established as a treatment for intestinal inflammation, and its broad effects on T cells and other lymphocyte populations indicate that therapeutic activation of this pathway is likely to be highly cell-type- and disease-context-dependent (115–118).

### 4.2 Small-molecule, dietary, and metabolite-based interventions

Orally administered small molecules and dietary compounds may provide opportunities for convenient dosing and local intestinal exposure; however, their pharmacokinetics, cell-type specificity, and systemic safety require compound-specific evaluation. Retinoid-, AhR-, and vitamin D-related interventions have shown the potential to modulate ILC3-associated functions in experimental settings, but most supporting evidence remains preclinical, and selective targeting of ILC plasticity has not yet been established.

Retinoic acid, a bioactive metabolite of vitamin A, has been investigated as a potential modulator of barrier-supportive ILC3 programs. *In vitro*, retinoic acid, together with IL-2, IL-1β, and IL-23, promoted the acquisition of IL-22-producing ILC3-like characteristics by human CD127^+^ ILC1s (14). In experimental intestinal inflammation, retinoic acid enhanced intestinal homing and IL-22 production by ILC3s and was associated with reduced disease severity (119, 120). These findings support further investigation of vitamin A- or retinoic-acid-based interventions, but the evidence remains preclinical. Because retinoic acid regulates multiple immune and epithelial-cell populations, its effects cannot currently be attributed specifically to ILC plasticity.

Dietary AhR ligands represent another potential strategy for modulating intestinal ILC3-associated functions. Compounds such as indole-3-carbinol, curcumin, and resveratrol can activate AhR-associated pathways that support intestinal ILC3 maintenance and IL-22 production (121, 122). Dietary supplementation with AhR ligands increased intestinal ILC3 abundance and IL-22 production and alleviated DSS-induced colitis in mice (123). However, their oral bioavailability, intestinal exposure, effective dose, off-target activity, and systemic safety differ among compounds and require individual evaluation. Moreover, direct evidence that these interventions selectively regulate ILC plasticity in patients with IBD remains unavailable.

Vitamin D and its active metabolite, 1α,25-dihydroxyvitamin D_3_, may also regulate intestinal ILC3 functions, but the available findings are species dependent. Vitamin D/VDR signaling promoted colonic ILC3 proliferation and influenced IL-22 production in mice, whereas 1α,25-dihydroxyvitamin D_3_ reduced IL-23 receptor signaling and altered cytokine production in human NKp44^+^ ILC3s (124, 125). These divergent findings indicate that the effects of vitamin D-related interventions cannot be directly extrapolated across species or assumed to be uniformly protective in human IBD.

Collectively, cytokine-directed therapies, retinoic acid, dietary AhR ligands, natural compounds, and vitamin D-related interventions illustrate the potential of manipulating ILC-associated pathways. However, most evidence remains indirect or preclinical, and the lack of ILC-specific delivery and reliable biomarkers currently limits clinical translation. These limitations are discussed in the following section.

### 4.3 The challenge of precise targeting without compromising host defense

Translating ILC-directed interventions into clinical practice faces three major challenges: limited treatment specificity, substantial ILC heterogeneity, and interindividual or disease-stage-dependent variation.

First, most pathways implicated in ILC plasticity are not restricted to ILCs. Cytokines such as IL-12 and IL-15 also regulate conventional NK cells, T cells, and other immune-cell populations, while ILCs themselves are broadly distributed across mucosal and lymphoid tissues (18). Systemic blockade or activation of these pathways may therefore alter protective immune responses outside the intestine and produce unintended immunological effects. For example, IL-12 blockade may reduce inflammatory ILC3-to-ILC1-like remodeling but can also impair Th1- and NK-cell-mediated host defense (126). Consequently, the clinical effects of existing cytokine-directed therapies cannot currently be attributed specifically to modulation of ILC plasticity.

Second, intestinal ILC populations are heterogeneous across anatomical sites, tissue compartments, and inflammatory states. Lamina propria ILC1s and intraepithelial ILC1s differ in phenotype and cytokine responsiveness, while the relative abundance of ILC1s, ILC2s, and ILC3s varies according to intestinal location and disease status (20). In active CD, expanded CD127^+^CD94^+^ group 1 ILC populations and transcriptionally intermediate ILC3–ILC1-like states further complicate the distinction between stable lineages and inflammation-induced phenotypes (65, 68). Recent single-cell studies also indicate that immune-cell composition differs substantially between the ileum and colon and between inflamed and less-inflamed tissue compartments (66, 67). These features make selective identification and therapeutic targeting of individual ILC states difficult.

Third, the relevance of individual ILC states varies according to disease subtype and stage. Increased intestinal ILC1 frequencies and reduced NKp44^+^ ILC3 frequencies are most consistently observed in active CD, particularly in inflamed terminal ileal tissue, whereas comparable directional ILC3-to-ILC1-like remodeling has not been clearly demonstrated in UC (20, 66). ILC composition may also differ between newly diagnosed and established disease and between inflamed and non-inflamed intestinal regions (26, 66, 67). These differences limit the feasibility of applying a uniform ILC-targeted treatment strategy to all patients with IBD.

A further translational limitation is that most detailed assessments of human intestinal ILC populations currently rely on mucosal biopsy or surgically obtained tissue samples (20, 26, 66–68). This dependence on tissue-based analysis restricts repeated monitoring of ILC states during treatment and remission. Future studies should integrate single-cell transcriptomics, spatial profiling, and longitudinal sampling to identify clinically accessible biomarkers of disease-associated ILC states. In parallel, intestinally localized delivery systems may help restrict therapeutic exposure to relevant tissue compartments, although their efficacy and ILC specificity require direct experimental validation.

Together, these challenges indicate that future therapeutic strategies should consider not only the direction of ILC plasticity but also the affected intestinal segment, tissue compartment, disease subtype, inflammatory stage, and potential effects on non-ILC immune populations.

## 5 Conclusions and outlook

ILC plasticity may contribute to context-dependent remodeling of the intestinal immune barrier in IBD. Rather than representing phenotypic change alone, these state transitions may reshape barrier function by altering epithelial repair, antimicrobial defense, cytokine output, and interactions among mucosal immune cells. Current evidence most strongly supports cytokine-dependent remodeling between ILC3 and ILC1-like states in CD, whereas a comparable directional transition has not been established in UC. ILC2 and ILC3 subsets also exert context-dependent reparative and inflammatory functions, but changes in their abundance should not be interpreted as direct evidence of lineage conversion.

Several key gaps remain. Human studies are largely cross-sectional and lack definitive lineage-tracing evidence, making it difficult to distinguish plasticity from recruitment, proliferation, survival, or selective loss of individual subsets. The extent to which epigenetic and metabolic reprogramming causally drives ILC-state transitions in human IBD also remains unclear. In addition, whether analogous directional remodeling occurs in UC, and how ILC states vary across intestinal segments, disease stages, and treatment responses, require further investigation.

Existing cytokine-directed therapies and experimental metabolic or small-molecule interventions may indirectly influence ILC states, but none currently targets ILC plasticity selectively. Future studies integrating longitudinal sampling, single-cell and spatial profiling, clinically accessible biomarkers, and disease-subtype stratification may help determine whether ILC-state modulation can be translated into safe and effective therapeutic strategies.
